# Inflammation in COVID-19 and the Effects of Non-Pharmacological Interventions during the Pandemic: A Review

**DOI:** 10.3390/ijms232415584

**Published:** 2022-12-09

**Authors:** Vicente Javier Clemente-Suárez, Álvaro Bustamante-Sanchez, José Francisco Tornero-Aguilera, Pablo Ruisoto, Juan Mielgo-Ayuso

**Affiliations:** 1 Faculty of Sports Sciences, Universidad Europea de Madrid, Tajo Street, s/n, 28670 Madrid, Spain; 2 Research Center in Applied Combat (CESCA), 45007 Toledo, Spain; 3 Department of Health Sciences, Public University of Navarre, 31006 Pamplona, Spain; 4 Department of Health Sciences, Faculty of Health Sciences, University of Burgos, 09001 Burgos, Spain

**Keywords:** COVID-19, inflammation, physical activity, nutrition, physiotherapy, psychology, life patterns

## Abstract

Severe acute respiratory syndrome coronavirus 2 (SARS-CoV-2) caused the coronavirus disease 2019 (COVID-19) pandemic that hit the health systems worldwide hard, causing a collapse of healthcare systems. One of the main problems of this new virus is the high inflammatory response it provokes, which is the cause of much of the symptoms. Different pharmacological approaches tried to stop the advance of the pandemic, but it seems that only vaccines are the solution. In this line, different nonpharmacological approaches have been made in order to improve symptomatology, contagion, and spread of COVID-19, the principal factors being the physical activity, nutrition, physiotherapy, psychology, and life patterns. The SARS-CoV-2 virus produces a disproportionate inflammatory response in the organism of the guest and causes complications in this that can end the life of the patient. It has been possible to see how different nonpharmacological interventions based on physical activity, nutritional, psychological, and physical therapy, and lifestyle changes can be functional tools to treat this inflammation. Thus, in the present review, we aim to provide an overview of the role of inflammation in COVID-19 and the nonpharmacological interventions related to it.

## 1. COVID-19 and Inflammation

SARS-CoV-2 is a contagious disease with origin in Wuhan, China [1]. Spreading around the world and affecting the population worldwide, with more than 110 million confirmed cases and more than 2.5 million deaths on its peak, has led to the COVID-19 pandemic situation [2]. 

Coronaviruses typically infect only the upper respiratory tract and cause minor symptoms [3]. However, the SARS-CoV-2 can replicate in the lower respiratory tract and cause pneumonia [4] and lead to a state of hyper-inflammation, known as the “cytokine storm” and thrombotic manifestations in different organs, which can be fatal [5]. In this line, there are two pathophysiological processes of SARS-CoV-2 differentiated and related to each other by the inflammatory and immune responses [6]:

1. Phase I: direct cytopathic effect that derives from the viral contagion that will prevail onset of the disease.

During this phase, there is a controlled death and injury of virus-infected cells and tissues as part of the virus replicative cycle [7]. Viral infection and replication in airways could cause high levels of virus-linked pyroptosis [8], a highly inflammatory form of programmed cell death common amongst cytopathic viruses [9]. The segregation of the cytokine interleukin-1β (IL-1β), the cytokine released during pyroptosis [10], together with the detection of pathogen-associated molecular patterns (PAMPs) and damage-associated molecular patterns (DAMPs) by alveolar epithelial cells and alveolar macrophages induces a local inflammation involving increased secretion of the proinflammatory cytokines and chemokines interferon gamma (IFN-γ), monocyte chemoattractant protein-1 (MCP-1), interferon gamma-inducible protein-10 (IP-10), interferon gamma (IFN-γ), and IL-6 [11]. Cytokines and chemokines secreted attract monocytes and T lymphocytes but not neutrophils into the infected site [12]. This recruitment of immune cells to the pulmonary system may explain the lymphopenia and increased neutrophil–lymphocyte ratio seen in around 80% of COVID-19 patients [13]. Most patients can suppress the infection with the recruited cells aforementioned and recover from the disease. However, some cases evolve differently, and a dysfunctional immune response ensues with an uncontrolled cytokine secretion and widespread lung inflammation, leading to phase II [4].

2. Phase II: nonregularized inflammatory replication of the host cell, the same as prevails in the later phases. The interleaving of these two pathophysiological phases refers phenotypically in the progress of three stages of the illness [14].
  (a) Stage I (early phase): it results from the replication of the virus that establishes the cytopathic sequela and acceleration of the innate immune response, identified by presenting mild symptoms, such as fever, cough, and myalgia (included in phase I).
  (b) Stage II (pulmonary phase): the result of the activation of the adaptive immune response, which results in a decrease in the viremia but indicates an inflammatory cascade causing tissue damage. It is determined by a worsening of the respiratory condition that can cause acute respiratory failure, accompanied by worsening lymphopenia and moderate increase in transaminase C.
  (c) Stage III (hyperinflammatory phase) represented by sudden multi-organ insufficiency, with frequent deterioration of pulmonary capacity, the result of an uncontrolled immune response that establishes the cytosine storm syndrome, which resembles secondary hemophagocytic lymphohistiocytosis [15] and causes 28% of fatal COVID-19 cases [16].

Among these stages, patients may exhibit higher blood plasma levels of IL-2, IL-7, IL-10, granulocyte colony-stimulating factor (G-CSF), IP-10, MCP1, macrophage inflammatory protein 1α (MIP1α), and tumor necrosis factor (TNF) [10,16]. IL-6 levels continue rising as the disease develops in these patients and are higher in non-survivors than survivors [17]. Furthermore, there exists a highly inflammatory monocyte-derived Ficolin-1+ (FCN1+) macrophage population in the bronchoalveolar lavage fluid [18] and a significantly higher percentage of CD14+ and CD16+ inflammatory monocytes in peripheral blood of severe SARS-CoV-2 cases [17]. These cells secrete more inflammatory cytokines MCP1, IP-10, and MIP1α that contribute to the ongoing cytokine storm and the exacerbated inflammatory response [4]. In addition to the direct damage dealt by the virus, an unrestrained inflammatory response can itself mediate damage to the lung through excessive secretion of proteases and reactive oxygen species, resulting in desquamation of alveolar cells, hyaline membrane formation, and pulmonary edema [19], limiting gas exchange efficiency, breathing difficulty, and low blood oxygen levels, and leaving the lungs vulnerable to secondary infections [20]. This uncontrolled inflammatory response not only affects local tissues, but it has ripple effects across the body. Elevated levels of cytokines, such as TNF, can cause septic shock and multi-organ failure, which may result in myocardial damage and circulatory failure.

Regardless of the stage and phases, patient’s symptomatology may be very diverse, regarding previous pathophysiology and comorbidities of the patient and his inflammatory and immunological response, which can be [20]:

Clinical mild symptoms: fever <38° without cough, dyspnea, or wheezing, no comorbidities, and without evidence of lung parenchyma.

Clinical moderate symptoms: dyspnea, high fever >38°, and gastrointestinal symptoms as nausea, vomiting, and diarrhea.

Clinical severe symptoms: respiratory rate >30 min, qSOFA score 2 or more, SPO2 ≤93% at rest, PaO2/FiO2 <300 mmHg, bilateral parenchymal involvement >50% in 24–48 h while experiencing confusion, agitation, and restlessness.

Clinical critical symptoms: respiratory failure/acute respiratory distress syndrome (ARDS), septic shock and multiple organ dysfunction syndrome (MODS).

SARS-CoV-2 employs the highly glycosylated spike protein (S) in order to penetrate cells and easily bind to the angiotensin-converting enzyme receptor 2 (ECA2). This enzyme is found indicated in type II alveolar cells. The RNA of the virus enters the cells from the nose and lower respiratory tract to be transferred to viral proteins. This has been linked as the major entry point for SARS-CoV-2. However, in the pathophysiology of COVID-19, ECA2, through the production of angiotensin 1-7, has anti-inflammatory qualities and very important antifibrotic properties, effects that occur through the receptor MAS [21]. Thus, the ECA2–ANG1-7–MAS receptor axis and the ECA–ANGII–ATA 1 receptor pathway can be seen as opposite but very complementary and where balance is necessary for optimal health [22]. 

Therefore, to restore or maintain the natural balance between the receiving axis ECA2–ANG1-7–MAS and the ECA–ANGII–ATA 1 receptor pathway and maintain the susceptibility and risk of COVID-19 physiopathology, physical exercise may be an optimal stimuli approach [23]. Physical exercise can increase the ACE2–ANG1-7–MAS receptor axis by inhibiting simultaneously the ACE–ANGII–ATA 1 receptor [24]. Yet, if, as authors suggest, severe and critical COVID-19 symptomatology leads to long-term lung damage [24], in cardiopulmonary rehabilitation, exercise should be mandatory, since, as mentioned before, the activation of the ECA2–ANG1-7–MAS receptor with physical training reduces pulmonary fibrosis [22]. Furthermore, muscle contraction during exercise is thought to be the main trigger for overexpression of IL-6 [25]. Its production in chronic inflammatory rheumatic and musculoskeletal diseases is induced by macrophages in the presence of TNFα, activated by the nuclear factor-kB (NF-kB) pathway [26]. However, IL-6 overproduction during muscle contraction appears to have no inflammatory effects, since it occurs without the presence of TNFα or NF-kB activation and is accompanied by other anti-inflammatory events [27]. TNFα inhibitors increase during and post-exercise [26]. IL-1 receptor antagonist is also produced during exercise and remains elevated immediately after [26], while IL-1b and TNFα inflammatory response mediators, remain suppressed during exercise [26] and proinflammatory M1 macrophages decrease, while anti-inflammatory M2 macrophages increase during exercise in healthy people [27], also impacting on the expression of proinflammatory cytokines [28], thus suggesting the acute anti-inflammatory effect of exercise. Furthermore, chronical effects on inflammation linked to exercise are thought to be mainly mediated by adipose tissue reduction, as adipose tissue plays a capital role in stimulating the increase in inflammatory mediators [29].

In this line, observations from Wuhan at the start of the pandemic showed hypertension (30%), diabetes (22%), and coronary artery disease (22%), usually associated with obesity, as the main comorbidities in COVID-19 patients who required hospitalization [30]. Chronic inflammation, condition accompanying obesity, and metabolic syndrome produces an abnormal proinflammatory cytokine production of TNFα, IL-1, and IL-6 and increased acute phase reactants [31]. Due to this condition, the innate immune response in patients with obesity is altered, with an increased inflammatory response and abnormal T-cell response [32]. These conditions may lead to a deficiency in control of viral replication and a longer proinflammatory response with a poorer outcome, which may lead to hospitalization when contracting COVID-19 disease in patients with these previous comorbidities compared to healthy patients [17]. 

Furthermore, the acute and chronic inflammation caused by COVID-19 physiopathology may have long-term consequences in those that recover, as authors suggest [33], leading to chronic medical conditions, likely through neuroinflammatory mechanisms that can be compounded by an unhealthy diet [34]. In this line, a balanced and equilibrated diet with vitamin D, vitamin A, B vitamins (folate, vitamin B6, and vitamin B12), vitamin C, and the minerals, Fe, Cu, Se, and Zn will promote and contribute to the normal function of the immune system and the inflammatory response [35,36]. Yet, one diet synonymous of anti-inflammation is the Mediterranean diet, which is a diet, traditional in Mediterranean countries, characterized especially by a high consumption of vegetables and olive oil and moderate consumption of protein, and thought to confer health benefits [37,38], fulfilled with anti-inflammatory and immunomodulatory compounds, such as essential vitamins (C, D, and E) and minerals (zinc, copper, calcium, etc.) [39,40]. It also includes foods containing bioactive compounds, such as phenolic compounds; polar lipids; and peptides with potent anti-inflammatory effect, antithrombotic, and antioxidant properties, preventing inflammation and associated thrombotic and reactive oxygen species (ROS)-related complications [41,42]. Likewise, considering that the bulk of hospitalized COVID-19 patients suffer from malnutrition and deficiencies in vitamin C, D, B12 selenium, iron, ω-3, and medium- and long-chain fatty acids, highlighting the potential health effect of vitamin C and D interventions [41], the inclusion of a Mediterranean diet can be an adequate nutritional pattern, which provides protection against noncommunicable diseases and, potentially, against COVID-19 [37,43].

In addition to adequate levels of physical activity and nutritional habits to control the immunological and inflammatory response, an optimal psychological state is also essential. The immune system and inflammation are currently accepted as contributors to brain-related diseases in most neurological and psychiatric disorders. Immune system activation is reflected in abnormal levels of inflammatory cytokines in serum, plasma, and cerebrospinal fluid of patients with psychiatric and neuropsychiatric disorders, creating a sustained proinflammatory state, believed to be involved in the pathogenesis and pathophysiology of major mental illnesses [44]. Furthermore, controlling inflammation without immunosuppression has been hypothesized to improve antidepressant treatment outcomes [45]. Furthermore, stress activates the hypothalamic–pituitary–adrenal axis and the sympathetic branch of the autonomic nervous system, with the subsequent reduction in vagal tone, resulting in a homeostatic imbalance, which contributes to a proinflammatory state [46,47].

Other techniques and habits can also help regulate the immune and inflammatory response. In this line, nerve stimulation is an emerging field in modern medicine used to control organ function and re-establish physiological homeostasis during illness [48], with recent studies indicating therapeutic benefits in treating inflammation in noncommunicative diseases (arthritis, colitis, obesity, or diabetes) and other infectious disorders, such as septic shock and severe sepsis [49]. Recent noninvasive strategies for nerve stimulation, such as electroacupunture, are currently being used to control inflammation and prevent organ damage in inflammatory and infectious disorders and re-establish physiological homeostasis [50].

Whether as a preventive factor or as a treatment, correct lifestyle habits, controlled daily physical exercise, and adequate nutrition are the keys to controlling the immune and inflammatory response and keeping the pathophysiology and comorbidity of COVID-19 at bay.

## 2. Nonpharmacological Interventions

The present research aimed to highlight the principal nonpharmacological interventions in COVID-19. For this aim, the principal results of the actual literature in physical activity, nutrition, physiotherapy, psychology, and life patterns are presented in the following points. 

### 2.1. Physical Activity

Physical activities are divided into aerobic, resistance, and diary activity interventions to better understand the effect of these three different activities. 

Aerobic exercise impacts all the immune cells, improving the performance of natural killer cells, neutrophils, and macrophages, following moderate exercise. Innate and adaptive immune systems are enhanced after acute, transient, and long-term adaptations to physical activity in a dose–response relationship, with an increase in higher maximal oxygen uptake as one of the underlying factors to avoid COVID-19 symptoms [51,52]. Regular physical activity, through moderate-intensity aerobic training, has been regarded as a protective factor against the severity of COVID-19 relating to thromboinflammation and its complications. Regular exercise could be an adjuvant for the prevention and treatment of COVID-19 [53], since it fosters angiogenesis, an enhanced vascularization, and a decreased chronic inflammation [54]. 

Exercise immunology researchers have reported the acute and chronic effects of exercise in the immune system that are maximized by adaptive responses primarily based on improved immunosurveillance and reduced systemic inflammation [55]. The role of angiotensin-converting enzyme-2 (ACE2), which is the receptor for SARS-CoV-2, after a COVID-19 infection leads to the increase in ACE2 levels through pathological complications, leading to neurological and cardiovascular problems that could be avoided by physical exercise [56].

Vigorous exercise that performs an overload to the cardiovascular system has been reported to mobilize and redistribute effector lymphocytes (through catecholamines effect) that move from reservoirs—such as blood vessels, spleen, and bone marrow—to tissues and organs—such as the upper respiratory tract and lungs—and improve the immune surveillance [57]. The important relationship between cardiovascular fitness and health outcomes has been supported by research that investigated maximal exercise capacity (determined by the Bruce test) that proved to be inversely associated with the odds of being hospitalized due to COVID-19 infection [58]. The health status of the people prior to infection is of vital importance to predict the severity of the COVID-19 symptoms. Overweight, insulin resistance, poor dietary habits, and a lack of physical activity have been linked to a chronic low-grade inflammation due to an increased level of several proinflammatory cytokines. This chronic status rises the odds of contracting a COVID-19 infection with more hazardous symptoms [59]. 

Sports medicine and laboratory diagnostics suggest that moderate exercise can help to reduce inflammation, to maintain thymic mass, and to enhance immunosurveillance [60]. Intense exercise, if performed inadequately, can open a window of immune system malfunction just after exercise, in which airborne infection could be easier [61]. In conclusion, it has been widely reported that moderate intensity activity can be prescribed as the preferred nonpharmacological way to prevent COVID-19, while vigorous exercise should be better explored. A careful assessment of each patient should be previously considered [62], since high-intensity exercise could increase thrombotic risk [63]. 

If we analyze recovery strategies after being infected by COVID-19 with hospitalization, the higher aerobic capacity and aerobic capacity change during recovery may be linked with a higher work activity level before the illness. Continuing physical activity, even at home, will promote recovery after the illness [64]. According to research, there are three mechanisms that protect from COVID-19 infections and increase the aerobic performance of patients: improved function of immune cells; antibiotic, antioxidant, and antimycotic effects in lungs that improve respiratory functions; and an enhanced immune function that could improve the barriers to stop the progression of this disease [65]. Since COVID-19 rapidly affects aerobic performance of young adults, a previous aerobic capacity could help to act as a barrier against its symptoms [66,67]. 

#### 2.1.1. Resistance

Resistance training, such as eccentric training, could be useful as a coadjuvant treatment to avoid COVID-19 symptoms. The temporal stress induced by resistance training may contribute to a long-term improved immune system. When exercise is repeated on a regular basis, it may act as a natural vaccine against viral infections, such as COVID-19, and should be further researched to control and prevent this disease [51]. To compensate for the usual activities during the lockdowns of a pandemic, there has been recommended at least two to three resistance sessions per week to compensate for the decreased mobility demands of the population [68]. Performing resistance exercise at home has been also reported to be necessary to avoid airborne infections and to maintain healthy resistance loads for the body; lifting and carrying, lunges, stair climbing, stand-to-sit and sit-to-stand using house items, squats, and sit-ups could be useful exercises to perform at home [69,70]. 

It seems that concurrent training is less effective than only aerobic or resistance training, although it is also capable of improving immune function. Moderate interval-based aerobic cycle endurance combined with resistance training had a good effect to achieve individually tailored goals for physical performance and health-related quality of life after COVID-19 hospitalization, compared to other approaches [71].

Specific resistance training based on respiratory muscles (that are the most affected by COVID-19 disease) have been developed: inspiratory muscle resistive training and respiratory muscle isocapnic hyperpnoea training. Both methods try to develop inspiratory overload to stimulate the respiratory muscles, subsequently improving respiratory ability. It is generally recommended to perform 50–100 resisted breaths daily, 5 days a week [72].

For healthy adults, home-based endurance and resistance exercise can reduce the detrimental fitness and health effects of detraining and could help to boost the immune system. For athletes, it is recommended to continue training but to avoid exhaustion to not compromise the immune system. For the elderly, a mixture of resistance, strength, and balancing exercise is recommended to avoid muscle loss and to enhance psychological functioning [73,74]. However, in a sample of young adults, upper extremity and trunk strength were not affected by a COVID-19 infection, suggesting that the most affected areas are related to respiratory function [66]. However, the importance of strength training for the breathing muscles [60] during the recovery period of older patients after an acute COVID-19 infection has been highlighted [75,76]. Whole-body vibration has been also explored to propose a different way to stimulate muscles and reduce inflammation in patients who have acute symptoms that prevent them from using traditional exercise as a countermeasure [77].

#### 2.1.2. Diary Physical Activity

Physical activity is one of the fundamental pillars in nonpharmacological interventions for the treatment of acute and chronic inflammatory processes. Within physical activity, the accumulated daily, that is, daily physical activity, is the key, since it represents the sum of physical activity per week and per month, that is, the chronic load of physical exercise, which is what we are looking for in intervention processes post-positive chronic load. In this line, increasing evidence suggests that the reason why only certain chronic diseases predispose to harmful symptoms is because they are linked with a proinflammatory state and an imbalance between the proinflammatory angiotensin converting enzyme-1 (ACE1) and anti-inflammatory ACE2 axes. Many studies have demonstrated that aerobic exercise can quickly reverse these links, regardless of one’s age or sex. Although regular exercise would not reduce one’s risk of becoming infected with SARS-CoV-2, it would reduce one’s risk of experiencing severe disease symptoms [78].

Thus, a regular physical activity practice acts as a modulator of the immune system, which helps to obtain both a lower infection incidence, acute symptoms, and mortality rates in viral infections, such as COVID-19. During and after physical exercise, pro- and anti-inflammatory cytokines are released, preventing inflammation and acting as an adjuvant booster of the immune system to avoid damage caused by COVID-19 symptoms [79]. Moreover, frequent moderate exercise (at least 3–5 days a week) is associated with better mental and physical well-being and a lower prevalence of COVID-related allostatic overload compared to those who lived a sedentary life during the COVID-19 pandemic. Frequent moderate exercise was associated with improved well-being, subjective physical health, and decreased somatization [80]. 

The importance of a consistent diary physical activity has been highlighted, since meeting physical activity guidelines (more than 150 min a week of physical activity) has been associated with a lower chance for severe COVID-19 outcomes among adult patients [81]. Apart from the benefits of physical activity on its own, regular physical activity has been previously reported to boost vaccination programs due to an increase in T cells and neutralizing antibodies. More research is needed in the case of acute exercise to boost vaccine responses, where there have been inconsistent findings in terms of the benefit to adding exercise bouts to vaccines to frail adults [82]. The risks of lockdowns during a pandemic also imply a less active lifestyle and a need to review the usual physical activity guidelines; to compensate for this loss, a weekly increase in physical activity from 150 min to 200–400 min aerobic exercise during lockdowns is suggested [68]. Moderate-intensity physical activity between 150 and 300 min per week has been consistently linked with enhanced immunosurveillance and lower respiratory illness hazard. On the other hand, overtraining and exercising while infected with a respiratory pathogen have been linked to immune dysfunction and an elevated risk for respiratory illness. This model can be applied to COVID-19, with the high likelihood that risk of morbidity and mortality is moderated in lean, physically active individuals of all ages. Some psychological disorders that have been reported after the pandemic quarantine, such as fear, anxiety, and post-traumatic stress disorder, can depress immune function. Exercise training increases endorphin, dopamine, and serotonin, all of which can contribute to enhancing immune function [72]. 

Not only are responses to diseases heterogeneous, but also responses to exercise may be different for different populations. Physical fitness, the characteristics of the training, or the individual pre-existing immunological characteristics can have an influence. Prior physiological and psychological conditions need to be considered to provide tailored immunological responses [83]. Continuous moderate and adapted exercise may be doubly beneficial in type II diabetes mellitus and cardiovascular diseases for preventing inflammation and viral respiratory infection (including COVID-19). Hypertension, diabetes, and cardiovascular disease are pre-existing comorbidities that increase the risk of COVID-19 symptoms due to their systemic inflammation. Exercise training programs would enhance immune protection for patients with these diseases [84], while they foster immunomodulation through the recommended guidelines of moderate physical activity [85].

### 2.2. Nutrition Interventions

The apparition of COVID-19 has made more and more research look for anti-inflammatory therapies that can buffer the cytokine storm in order to reduce the severity of this infection [5]. As in other serious infections, diet, exercise, and healthy lifestyle preferences can reduce the risk of development and could improve the prognosis of COVID-19 because they may be able to control the activity of inflammatory intermediaries [85]. In contrast, an unhealthy diet and lifestyle is associated with low-grade inflammation and increased oxidative stress, which could favor the spread and severity of COVID-19 [5]. In this sense, there is enough evidence to indicate that the consumption of some foods and nutrients affect the functioning of our immune system [86]. Therefore, although the difficulty of the interaction (nutrition and immunology) requires more research at present, it is recommended to ensure the recommended daily allowance (RDA) for those nutrients that are considered important for the proper functioning of immune functions [37].

A diet synonymous with anti-inflammatory properties is the Mediterranean diet, characterized by a high dietary intake of fruits, vegetables, olive oil, whole grains, and nuts, with a low/moderate consumption of fermented dairy products, fish, poultry, wine and, finally, a low consumption of processed and red meats [87]. A diet that conforms to these proportions of these foods is associated with anti-inflammatory and immunomodulatory nutrients, such as vital vitamins (A, B, C, D, and E) and minerals (copper, selenium, zinc, etc.), which affect the nutritional status of a person [39]. In this sense, it has been shown that higher adherence to the Mediterranean diet may be related with a lower risk of suffering from COVID-19 [88]. These results are in line with an ecological study of 23 countries where a negative association was also observed between adherence to the Mediterranean diet and deaths related to COVID-19 when adjusted for factors of well-being and physical inactivity [89].

The most characteristic food of the Mediterranean diet is olive oil [90], characterized by being a key bioactive food due to its high nutritional quality and, in addition to its particular composition in monounsaturated fatty acids (MUFA) and polyunsaturated fatty acids (PUFA), it is an important source of polyphenols (REF). The contemporary knowledge available on the beneficial effects of olive oil and its phenolic compounds, in particular, its biological properties and its antioxidant capacity against immune-mediated inflammatory responses [91] are a potential candidate to act against SARS-CoV-2 [92].

Fish and their oils are another important food group within the Mediterranean pattern. Its content in omega-3 polyunsaturated fatty acids (PUFA) could positively affect the progression and severity of COVID-19 [76,93]. PUFAs have anti-inflammatory properties through various cellular mechanisms, including depletion of proteins that play a central role in COVID-19-induced cytokine storms [94]. Furthermore, PUFAs have been shown to produce less pulmonary neutrophil infiltration and less pulmonary permeability in acute respiratory disease (ARDS), a well-known complication of COVID-19 [30]. In this sense, supplementation with 1 capsule/day with omega-3 (400 mg EPA and 200 mg DHA) enhanced the levels of various respiratory and renal function parameters in critically ill patients with COVID-19 [94].

Another group of high consumption foods in the Mediterranean pattern is that of fruits and vegetables. Although there are no studies that associate the intake of this food group and the suffering of COVID-19, an inverse relationship has been demonstrated between the intakes of fruits and vegetables with respiratory [95] and inflammatory conditions [96], two of the effects of COVID-19. This benefit may be due to its micronutrient profile that can exert an antioxidant, anti-inflammatory action, and other beneficial effects on the suffering of COVID-19 [97]. These micronutrients included in the foods of a healthy diet pattern, such as the B vitamins, vitamin C, D, and E, and the minerals Se, Cu, and Zn, are important for proper immune function [76]. Therefore, although in many cases there is no direct information on their role in COVID-19, it is plausible to believe that an adequate amount of these could potentially favor the prognosis of this disease by increasing resistance to infection [76,98].

In this line, although the role of group B vitamins, present in foods of animal and vegetable origin, in reducing inflammation and respiratory difficulties in patients infected by COVID-19 has not been demonstrated, their individual functions on the immune system make it a group to be reckoned with [99]. Adequate levels of thiamine (vitamin B1) are likely to aid adequate immune responses of antibodies (T cells) during SARS-CoV-2 infection [99]. For its part, riboflavin (vitamin B2), together with ultraviolet light, could alleviate part of the risk of transmission of COVID-19 through blood contact [100]. Niacin (vitamin B3) could be used as a complementary treatment for patients with COVID-19 because it is a basic component of NAD that has immunomodulatory properties by reducing proinflammatory cytokines [101]. Furthermore, it decreases neutrophil infiltration and shows an anti-inflammatory effect in persons affected with lung injury and avoids lung tissue damage [102]. Supplementation with pyridoxal 5′-phosphate (PLP), an active form of vitamin B6, can help mitigate COVID-19 symptoms by regulating immune responses, decreasing proinflammatory cytokines, maintaining endothelial integrity, and preventing hypercoagulability [99]. It has also been suggested that folic acid could be beneficial for the treatment in the early stages of respiratory disease associated with COVID-19 because it was able to inhibit furin, an enzyme associated with bacterial and viral infections, preventing the binding of the SARS-CoV-2 spike protein, preventing cell entry and virus renewal [103]. Lastly, vitamin B12 supplements have been shown to have the potential to reduce organic symptoms and damage related to COVID-19. In this sense, supplementation with 500 μg of vitamin B12, 1000 IU of vitamin D, and magnesium reduced the severity of COVID-19 symptoms and the need for oxygen and intensive care [104].

Vitamin C, present in fruits and vegetables, can prevent the progression of mild to severe symptoms in patients with COVID-19 [105]. It has been hypothesized that the use of ascorbic acid could reduce SARS-CoV-2 infection through the ability to stimulate the immune response, along with decreasing the severity of the virus-mediated inflammatory response [106]. In this sense, supplementation with high doses of vitamin C has improved oxygen support in patients [107] and showed reductions in inflammatory markers and in the risk of mortality in hospitalized patients [108]. So, high-dose vitamin C may be a promising therapy for COVID-19.

Vitamin E, present in vegetables and vegetable oils, acts as an antioxidant and protects cell membranes, including those of immune cells, from lipid peroxidation [109]. In addition, vitamin E levels have been shown to decrease in cases of influenza infection, and supplementation with vitamin E reduces the severity and duration of this illness [110]. In the same context, a meta-analysis showed that vitamin E reduced the levels of C-reactive protein (CRP), an element used to identify inflammations or infections in the body [111]. A combination of vitamin E and C has recently been proposed to improve cardiac lesions in critically ill COVID-19 patients, further showing its role in COVID-19 disease [112].

Vitamin D is an essential nutrient, whose plasma levels have been positively associated with lower severity in COVID-19 patients because it acts as a modulator of the immune system, providing an effective physical barrier and strengthening both adaptive and innate immunity [113]. Although this relationship is fully confirmed, there are few intervention studies that show the effects of vitamin D supplementation on the immune system of COVID19 patients. In this sense, although a single oral dose of 200,000 IU of vitamin D3 did not significantly reduce the length of hospital stay of COVID-19 patients [114], a dose of 60,000 IU for 8 or 10 days of vitamin D led to a significant reduction in the inflammatory markers associated with COVID-19 without showing side effects [115]. 

On the other hand, selenium, a mineral present in fish, seafood, red meat, chicken, eggs, and cereals, may play a role in the prevention of COVID-19 due to its antioxidant function, since it is a structural component of a family of antioxidant enzymes [116]. In parallel, selenium participates in a critical step in the immune response; therefore, selenium deficiency is associated with an increase in inflammatory molecules [11]. Although selenium is found in widely used foods, 42% of hospitalized COVID-19 patients have developed selenium deficiency [85]. In this sense, a supplementation with 1.0 mg/day of intravenous selenium for 10–14 days improved plasma selenium levels that were inversely correlated with inflammation markers in critically ill patients due to COVID-19 [117].

A mineral related to selenium is copper, which we can find in certain fruits and vegetables, as well as oysters and other shellfish. Copper deficiency is related to an increase in the rate of infections and mortality from COVID-19 [118]. Survivors of COVID-19 have been observed to show higher mean serum copper concentrations than those who died [119]. Likewise, a positive linear correlation was observed in total serum copper and selenium concentrations in all patient samples analyzed. These results would indicate that supplementation with these micronutrients in patients with deficiencies of the same can positively influence the prognosis of COVID-19, since it can prevent oxidative DNA damage and reduce inflammatory markers due to the fact that several cuproenzymes control the redox state and support the immune system [119].

Zinc also has anti-inflammatory and antioxidant effects, in addition to inhibiting the activity and replication of some viruses, such as the coronavirus (SARS-CoV-1) [120]. Although its deficiency is not very common, since we can find it in meat, fish, and seafood, it has been observed that deficient levels of zinc seem to increase the susceptibility to infections of the respiratory system that can be mitigated with supplementation with zinc [121]. On the other hand, zinc may mediate the beneficial effects of chloroquine, a drug that is widely used against COVID-19 because this drug increases intracellular levels of Zn^2+^ [19].

In conclusion, following a healthy dietary pattern, such as the Mediterranean pattern, will include foods (olive oil, fish, fruits, and vegetables) whose micronutrient profile will allow oxidative stress and high functionality of the system to be kept at bay, important elements to reduce the risk of serious conditions in patients infected by COVID-19.

### 2.3. Physiotherapy Intervention

COVID-19 reduces lung compliance and causes significant changes in lung function, with hypoxic and cardiovascular effects. These changes require management with physical therapy and oxygen therapy and ventilatory support in these patients. Survival and critically ill patients are often associated with severe dysfunction and poor health-related quality of life. Early physiotherapy and community-based rehabilitation of COVID-19 patients have recently been identified as essential therapeutic tools and have become an important evidence-based element in the management of these patients [121]. 

Patients usually present with a debilitated physical condition because of the disease, which reduces their exercise capacity, especially when they present with fever, dyspnea, myalgia, and fatigue, the debilitated physical condition being the result of prolonged mechanical ventilation and immobilization [122]. 

A variety of techniques and modalities of early physiotherapy in intensive care unit are suggested by clinical research, but, principally, chest physiotherapy and rehabilitation of respiratory muscles presented evidence that supports or partially supports this. Chest physiotherapy may improve respiratory function and quality of life in people with COVID-19, especially after discharge. Some patients with a productive cough may benefit from airway hygiene procedures and techniques to stimulate coughing [123]. However, be aware that chest physiotherapy is an individual treatment based on the patient’s specific symptoms. So, if a patient has symptoms that could benefit from chest physiotherapy, it can be given while the patient is closely monitored for side effects [124]. 

After the post-intubation and the discharge, interventions oriented principally to recover respiratory muscles have been applied. In this line, the most effective techniques were the rehabilitation involving respiratory muscle training, cough exercise, diaphragmatic training, stretching exercise, and home exercise [125]. These rehabilitation services are presented as essential, highlighting the necessity to continue this service during the pandemic and after it finishes, since they are an essential component of high-value care offered for individuals across the lifespan to optimize physical and cognitive functioning to reduce disability [126].

### 2.4. Psychology Interventions

Quarantine and lockdown measures in the contexts of the COVID-19 pandemic have limited mental health interventions, presenting a double challenge: first, to prevent increased risks in mental disorders; and second, to treat and mitigate mental health interventions to patients and health professionals [127]. 

Evidence suggests that COVID-19 is associated with an increased risk of developing a mental disorder and poor mental wellbeing and mediated by psychosocial factors, such as socioeconomic status [127]. The most vulnerable groups of negative mental effects from COVID-19 are those with previous mental disorders, health care professionals, and older people [128].

However, though the label is understandable, valid situational responses to psychiatric “disorders” may result in stigma and a perceived need for medication, when providing information, normalizing, validation, kindness, empathy, or social support may be even more effective [128]. For example, mental health care for medical staff in China during the COVID-19 outbreak has been considered a template for best practice “refused any psychological help and stated that they did not have any problems” [129]. Indeed, COVID-19-related distress should not itself be considered a mental disorder.

To date, epidemiological data on the mental health problems and psychiatric morbidity of those suspected or diagnosed with the 2019-nCoV and their treating health professionals have not been available, and mental health care for the patients and health professionals directly affected by the COVID-19 epidemic has been under-addressed [130].

Furthermore, since epidemiological data on the mental health problems of COVID-19 patients or health professionals are not yet fully available, most recommendations of nonpharmacological interventions for the actual COVID-19 pandemic are based on mental health consequences and measures taken during the 2003 SARS outbreak. 

In general, most patients experienced emotions of fear of the consequences of infection and loneliness and stigma. From a stress model perspective, the perception of uncertainty and uncontrollability are core predictors of increased stress and, therefore, increased risk of mental disorders and worse health, including depression and suicidality [131]. Moreover, recent studies suggest an increased risk in drug use and drug-related harms [132]. Health care professionals have also reported increased risk of depression, anxiety, fear, and frustration [130].

However, most health professionals, even those working in COVID-19 units, fail to receive any training in providing mental health care. 

Some recommendations to prevent or mitigate the increased risk of mental health problems include: first, to communicate with patients and health professionals regularly with clear and accurate updates about COVID-19 and their progress, if necessary using smartphone- or internet-based communications to reduce uncertainty associated with stress and isolation. Second, provide regular screening for depression, anxiety, and suicidality, followed by formal psychological treatments when needed. Interestingly, it is worth mentioning that recent studies suggest that the most common trajectory for both anxiety–depression in COVID-19 is characterized by a resilient little-to-no psychological distress [133].

Both patients and health care providers would also benefit from raising awareness about the mental health impact of COVID-19 and increasing access to public mental health and reducing social isolation resulting from lockdown and social distance measures to face COVID-19. 

In summary, mental health should be part of an integral response to COVID-19, with long-lasting positive effects that may outlast the pandemic. In order to reach this goal, it is a priority to develop psychological interventions to meet the mental health problems in both COVID-19 patients and health care professionals [134]. However, for most patients and health workers, emotional and behavioral responses are part of an adaptive response to extraordinary stress, so non-psychological interventions based on the stress-adaptation model might be helpful [134]. Moreover, more funding for providing an adequate psychological intervention is needed [135], since coverage of interventions to prevent mental disorder or promote mental well-being is negligible and future research should focus on this area.

### 2.5. Life Patterns

In a landmark study published in Science 100 years ago, two main lifestyle or behavioral factors should be considered to precedent the Spanish Flu pandemic [136]: first, risk misperception put oneself and others in danger; second, self-isolation or social distancing as a measure to protect others is contra intuitive and less likely to be endorsed.

This is interesting because most current studies have focused on COVID-19 as a pandemic associated with infection with severe acute respiratory syndrome coronavirus 2 (SARS-CoV-2). However, this infectious disease interacts with psychosocial factors, such as social inequality and other noncommunicable diseases related with lifestyle patterns. Indeed, the aggregation of these lifestyle patterns and the background of social and economic disparity, which may exacerbate the adverse effects of COVID-19 suggest that COVID-19 is rather a syndemic than a pandemic [137]. In other words, framing COVID-19 as a syndemic highlights the important role of psychosocial factors (encompassing education, employment, housing, food, and environment) and lifestyle patterns. Therefore, solutions should include lifestyle-based interventions, beyond seeking purely pharmaceutical solutions.

Currently, the total number of people living with chronic diseases associated with poor lifestyles is growing; therefore, when facing COVID-19, we should consider risk lifestyles associated with hypertension, obesity, diabetes, cardiovascular, chronic respiratory diseases, and cancer.

Certainly, tobacco smokers are also at high risk of severe COVID-19 infection due to poor lung function, because COVID-19 transmits through salivary droplets and may cause severe lung pneumonia [138]. Moreover, it is a potential mode of transmission for the virus for both active and passive smokers, because it produces through exhaled smoke, coughing, or sneezing aerosols containing the virus [138]. Based on past public health crises, long-term alcohol consumption may increase 1.5 times due to distress [139]. Furthermore, people with substance use are at risk for contamination and socioeconomic changes caused by the pandemic will add to the classical difficulties in access to and adherence to treatment, certainly worsening their condition with COVID-19 [140]. Moreover, the risk of a variety of symptoms ranging from sleep disturbances to suicide may be exacerbated due to COVID-19 [125]. In fact, sleep abnormalities are a stand-alone risk factor for suicidal ideation, suicide attempts, and suicide death [141]. Finally, according to recent research conducted by the American Diabetes Association, obesity is a risk factor for greater COVID-19 severity [142]. It has been hypothesized that obesity commonly aggravates the severity of respiratory diseases in three pathways: first, due to underlying low-grade chronic inflammation and suppression of innate and adaptative immune response; second, by providing an altered microenvironment able to support the emergence of potentially pathogenic variants responsible for greater disease severity; and, third, severe obesity is associated with mechanical dysfunction, increasing the severity of lower respiratory tract infection or secondary infections [142]. Finally, compelling evidence supports the link between larger exposure to poor air quality or air pollution and 9.4 times more COVID-19 cases and 3.0 times more hospital admissions [143]. For example, in a recent study conducted in England, regional levels of NO_2_, NO, and O_3_ correlated with number of COVID-19 cases and deaths (144). Moreover, small increases in air pollution have led to a large increase in COVID-19 cases and mortality [144]. Therefore, exposure to poor air or pollution may increase vulnerability and worsen the prognosis of COVID-19 patients [145].

The perception of risk of COVID-19 has consequences in daily behaviors and lifestyle patterns. The usual “optimisms bias” may lead to underestimating the likelihood of contracting a disease such a as COVID-19 [146]. Moreover, many risk perceptions are driven by emotional information rather than factual information. Emotions may bias the way in which information is processed. Strong emotional reactions may lead to ignoring important numeric information, such as probabilities of contagion or number of deaths [147]. This has important implications in how media should inform about COVID-19. Moreover, the perception of risk or threat by COVID-19 increases the risk of intolerance and discrimination or punitive attitudes towards out-groups [148]. This is relevant to establish social norms that balance freedom and social distancing in the context of COVID-19.

Indeed, the role of information is key, and the COVID-19 pandemic has resulted in an increase in conspiracy theories, fake news, and misinformation. This is a serious problem because more people may isolate themselves or make pervasive lifestyle decisions, such as hostility toward groups seen as responsible for the virus. There is no doubt that coping with COVID-19 will benefit from strategies that aim to engage in effective communication to increase persuasion around public health [149]. 

Social distancing and self-isolation are a key measure when dealing with COVID-19. However, social connection also helps people to regulate their emotions and cope with stress and remain resilient during adverse times [150]. Unfortunately, loneliness and social isolation worsen the burden of stress and often produce deleterious effects on mental, cardiovascular, and immune health [150].

On the positive side, a sense of shared identity and mutual aid groups among the public have become widespread in response to COVID-19 [147]. This enhances collaboration and result from addressing the public in collective terms and by urging “us” to act for the common good [151]. By contrast, competition may lead to stocking up on supplies in preparation for potential self-isolation [147].

In summary, the global health crisis associated with COVID-19 places important psychological burdens on individuals and requires a large-scale lifestyle change. Previous authors have already highlighted why insights from social and behavioral sciences can be used to align behaviors with recommendations of epidemiologists and public health experts [152].

## 3. Conclusions

The SARS-CoV-2 virus produces a disproportionate inflammatory response in the organism of the guest and causes complications in this that can end the life of the patient. It has been possible to see how different nonpharmacological interventions based on physical activity, nutritional, psychological, physical therapy, and lifestyle changes can be functional tools to treat this inflammation.

## Data Availability

Not applicable.
